# A Data-Driven Adaptive Sampling Method Based on Edge Computing

**DOI:** 10.3390/s20082174

**Published:** 2020-04-12

**Authors:** Ping Lou, Liang Shi, Xiaomei Zhang, Zheng Xiao, Junwei Yan

**Affiliations:** 1 School of Information Engineering, Wuhan University of Technology, Wuhan 430070, China; louping@whut.edu.cn (P.L.); shiliang@whut.edu.cn (L.S.); May125z@whut.edu.cn (X.Z.); 2 Hubei Key Laboratory of Broadband Wireless Communication and Sensor Networks, Wuhan University of Technology, Wuhan 430070, China; 3 School of Mechanical and Electronic Engineering, Wuhan University of Technology, Wuhan 430070, China; reallylaugh@whut.edu.cn

**Keywords:** edge computing, industrial internet of things, data acquisition, adaptive sampling, linear median jitter sum

## Abstract

The rise of edge computing has promoted the development of the industrial internet of things (IIoT). Supported by edge computing technology, data acquisition can also support more complex and perfect application requirements in industrial field. Most of traditional sampling methods use constant sampling frequency and ignore the impact of changes of sampling objects during the data acquisition. For the problem of sampling distortion, edge data redundancy and energy consumption caused by constant sampling frequency of sensors in the IIoT, a data-driven adaptive sampling method based on edge computing is proposed in this paper. The method uses the latest data collected by the sensors at the edge node for linear fitting and adjusts the next sampling frequency according to the linear median jitter sum and adaptive sampling strategy. An edge data acquisition platform is established to verify the validity of the method. According to the experimental results, the proposed method is more effective than other adaptive sampling methods. Compared with constant sampling frequency, the proposed method can reduce the edge data redundancy and energy consumption by more than 13.92% and 12.86%, respectively.

## 1. Introduction

With the arrival of the era of industrial internet of things (IIoT) [1] and big data [2], more and more data can be collected and analyzed. Industrial big data [3] has become an important driving force of the new round of industrial revolution. As the most practical and highest frequency demand of industrial big data in the production process, data acquisition [4,5,6] is also used as one of the measurement standards of industrial field automation integration. Data collection is widely used in many fields such as signal detection, equipment monitoring, signal processing and instrument detection. Although the data collection system has different definitions according to different application requirements, the purpose of each system is to provide a worry-free environment for the construction of smart manufacturing systems [7] in the future. In industrial field monitoring [8], a series of problems pose challenges to the development of industrial big data, such as the various of industrial equipment, the complexity of monitoring data, the long duration of monitoring and the large amount of data accumulation. According to the Cisco Cloud Index [9], by 2021, there will be more than 50 billion terminal devices worldwide and the total amount of data generated by these devices will reach 847ZB per year, about 10% of which will need to be processed. In contrast, global data centers are expected to have only 2.6ZB of storage capacity and 19.5ZB of network traffic. If all the data on the edge side is uploaded to the cloud computing center for analysis and processing, it will cause a serious problem of insufficient network resources. This large amount of data also poses new challenges to the development of cloud computing [10,11].

The emergence of edge computing [12,13] technology provides an ideal direction for the development of industrial big data. Edge computing refers to the open platform that integrates the core capabilities of network, computing, storage and application at the edge of the network near the object or data source to provide edge intelligent services nearby. Through the embedded computing platform [14], the terminal equipment will be continuously intelligent. It can carry out all offline computing or part of the calculation locally, which provides the possibility for the realization of edge computing. With the deployment of the edge computing layer [15], basic computing and control can be applied to the edge of the network without being handed over to the cloud. Being closer to the user, the edge device can help the user filter and process confidential data [16] and desensitize it to the cloud. Because the process is realized in the edge computing layer, real-time field control and feedback can be carried out on the site, which greatly improves the processing speed and efficiency.

With the continuous development of China’s industry, energy shortage has become the focus of China’s economic development and social progress. In order to better promote the rational and full use of energy in China, promoting the green development of industry has become a standard of industrial production. More and more green products have entered industrial production. Due to some special circumstances, the sensors need to be powered by batteries in the monitoring of industrial environment. The battery-powered sensors can effectively solve the problem of field wiring and interference. For such battery-powered sensors, energy consumption from data collection is the main way to affect service time. Adaptive sampling reduces unnecessary data collection and unnecessary energy consumption which extends the service time of battery-powered sensing devices. Therefore, reasonable data collection is particularly important.

The nodes of industrial sensor networks usually adopt the data collection method with equal time interval when collecting data: Collect data periodically according to the preset time interval. This method completely ignores the characteristics of data changes. The data collection method, using equal intervals, may cause two extreme situations: (1) When the sampling object changes slowly, a large amount of redundant data is collected because the data collection interval is too small, which will occupy a large amount of storage space; (2) If the data collection interval is too long, the information of the sampling object will be lost, and some sampling information at rapidly changing times will be lost, resulting in serious degradation of original data quality. The sampling frequency should be decreased to reduce unnecessary information collection when the sampling object changes slowly. By increasing the sampling frequency when the sampling object changes rapidly, the value of the sampling object at the sensitive time can be observed more deeply and more subtly, thus reducing the distortion of the sampling object and improving the system efficiency. In the process of data acquisition, unnecessary data acquisition not only takes up a lot of memory space, but also wastes the energy of sensing equipment. Aiming at the problem of sampling distortion, edge data redundancy [17] and energy consumption caused by the constant sampling frequency of traditional IIoT, a data-driven adaptive sampling method based on edge computing is proposed in this paper. Through the transformation of the network edge layer, the data collected on the edge data acquisition platform according to the adaptive sampling method. Adaptive sampling can reduce the sampling distortion, the redundancy of the edge data and the energy consumption during the data collection process.

The remainder of this paper is organized as follows: Previous research on adaptive sampling are stated in Section 2. Section 3 elaborates the construction of edge data acquisition platform and the detailed method of data-driven adaptive sampling-based edge computing, including how to change the sampling frequency according to the adaptive sampling strategy. Section 4 demonstrates the experimental setup and provides some experimental results. Finally, Section 5 concludes the paper.

## 2. Related Work

The international organization for standardization has its own specific minimum standard frequency value for sampling objects in industrial sites. The change urgency of different sampling objects varies greatly, so different sampling objects should adopt different sampling frequencies to collect data. Long-term data collection results show that even the same sampling object will change gently in one time interval and rapidly in another time interval, which requires the sampling frequency of the same sampling object to be adjusted appropriately in the sampling process to cope with the change of the sampling object. Due to the problems existing in traditional data collection methods, a reasonable solution is to adopt adaptive sampling frequency [18], dynamically adjust the sampling frequency according to the changes of the sampling objects: Decreasing the sampling frequency and the amount of data collection when the sampling object changes gently; Increasing the sampling frequency and the amount of data collection when the sampling object changes rapidly.

There are some researches on sampling frequency adjustment. Ilaria Scarabottolo put forward a kind of adaptive sampling frequency method based on spectral change detection for low-power embedded devices [19]. By detecting the variation of the signal spectrum through the change detection test (CDT), the purpose is to extract the variation of the signal. Thus, the sampling frequency can be adjusted adaptively; Jinyou Xiao proposed a method of using transient boundary element analysis to realize adaptive sampling frequency in the frequency domain [20]. This method used the vector fitting method [21] to fit the frequency response function of the rational function and then solved it. However, the algorithm is relatively complex and the limitation of computing resources will make it difficult to implement the adaptive strategy in a large range; Yanlong Sun put forward an adaptive sampling method to solve the problem of underwater node energy constraints and unbalanced energy dissipation [22]. Through using double input and single output fuzzy logic controller from the adjustment sampling interval to minimize the sampling frequency and reduce the energy consumption of the information exchange. Tongxin Shu put forward, a kind of, applied to battery performance management based on data driven adaptive sampling algorithm [23]. The key parameters in the water were collected by the sensor and the sampling frequency was adapted according to the fluctuation of the parameters. By reducing the data sampling, data processing and transmission of energy, the battery life was effectively extended. However, this method cannot completely detect the moment of rapid change of the sampling object and the information of rapid change of the sample object that is not within the specified range will be lost. Some important information would be hidden at this moment; Wassim Drira designed an adaptive data collection scheme based on location perception in the vehicle network data collection system [24]. In this scenario, they continuously compared the latest sampling data with a set of historical data to determine the frequency of messages transmitted between the traffic management center and the vehicle. However, their method is mainly to reduce energy consumption by reducing the number of data uploads after data collection, but the energy of unnecessary data collection has been consumed. The proposed method is mainly to save energy by reducing the number of unnecessary sampling during data collection.

The above adaptive sampling methods can be roughly divided into two categories: Spectrum-based adaptive sampling method and parameter fluctuations adaptive sampling method. The research works promote the adaptive variable frequency data acquisition strategy to a certain extent. However, most research work do not consider the feasibility of complex algorithm implementing on edge devices and the rapid response of the system to the fluctuation of the sampling values, so a data-driven adaptive sampling method based on edge computing is proposed in this paper. By applying the method proposed in this paper to the sensor, the effect of extremum value on the result is reduced and the accuracy of the method is improved. Table 1 summarizes the advantages and disadvantages of the existing methods and the method proposed in this paper. The adaptive sampling method needs to continuously adjust the sampling frequency in the sampling process according to the latest sampling value. The data-driven method will help keep it up to date. By applying the method proposed in this paper to sensors, sampling distortion, data redundancy and energy consumption can be effectively reduced.

## 3. The Method of Data-driven Adaptive Data Acquisition Based on Edge Computing

The main idea of data-driven adaptive data acquisition based on edge computing is to dynamically adjust the interval time between the sampling points at the data acquisition node according to the changes of the sampling objects. The sampling interval will increase as the data changes smoothly, and decrease as the data changes violently. For the analysis of data collection and data-driven adaptive sampling frequency method mentioned above, an edge data acquisition platform [25] at the edge of the internet of things is established firstly. Then, a data-driven adaptive sampling method based on edge computing is proposed to analyze the function modules of the edge platform in the edge gateway section.

### 3.1. Edge Data Acquisition Platform

This section proposes a data acquisition platform based on edge computing. According to the flow direction of the IIoT field data, the data can be divided into two categories:

1. Uplink state data, data generated and collected during the production process of the product, including processing monitoring data, production environmental monitoring data and products quality feedback data.
2. Downstream control data, data received by production equipment, including control data and configuration data for industrial equipment. Aiming at the collection and feedback of IIoT field data.

**Figure 1:**
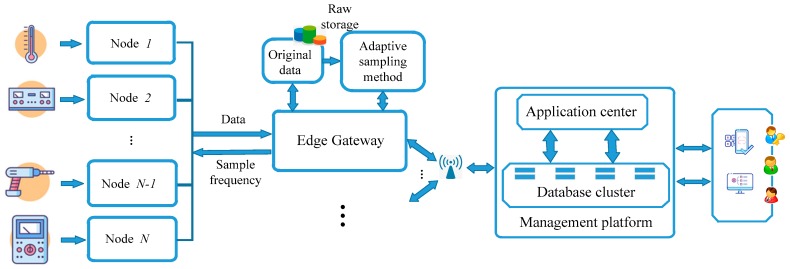
The structure of edge data acquisition platform.

The upstream data in the data acquisition platform is equipment processing data and environment monitoring data. The downstream data is configuration data, such as the sampling frequency of sensors. As shown in Figure 1, the acquisition platform mainly includes the following parts:

- Collection node: The collection node is used to collect data by various protocols on the industrial site. Through the sensing and collection of industrial field production data, the collection and transmission of equipment parameters and environmental data are realized.
- Edge gateway [26]: Edge gateway has the function of providing computing, storage, network and other infrastructure resources. Considering the complexity of communication connection among industry terminal equipment, edge gateway also requires the ability to have abundant interface/contracts. The edge gateway supports a variety of physical equipment protocol parsing and transformation, simple analysis, temporary storage and small batch data query. Edge gateway can transfer specific data to the management platform, realizing the communication between operation technology (OT) and information technology (IT).
- Management platform: The management platform is used to manage the edge gateway cluster, to set up the database cluster for the data uploaded by the edge device and to manage the data uniformly. The management platform provides a number of field-level applications to facilitate the management of production equipment. The long-term benefits, quantity and quality of information can be greatly improved through the establishment of the management platform.

Based on the above edge computing platform, the IIoT data collection system is realized. The system completes the analysis and storage functions of collected data on the edge side. It can also combine with the management platform to support more complex and perfect industrial application requirements.

### 3.2. Data-driven Adaptive Sampling Method

**Figure 2:**
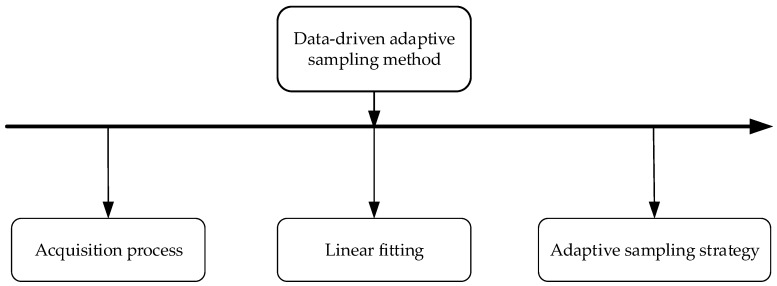
The process of data-driven adaptive sampling method.

Based on the above description of the edge data acquisition platform, we adopt data-driven adaptive sampling methods at edge gateway with infrastructure resources such as computing, storage and network. The data-driven adaptive sampling method is mainly divided into three stages. The process of the data-driven adaptive sampling method is shown in Figure 2. In the first stage, the acquisition process is established on the edge acquisition device. The latest collected data continuously drives and adjusts the parameters of the linear fitting in the next stage and the output of the adaptive sampling strategy. In the second stage, a linear fitting relationship is established based on the sampling data. In the third stage, the linear median jitter sum is calculated based on the established linear fitting relationship and the next sampling interval of the sensor is adjusted according to our proposed adaptive sampling strategy.

#### 3.2.1. Establishment of Acquisition Process

**Figure 3:**
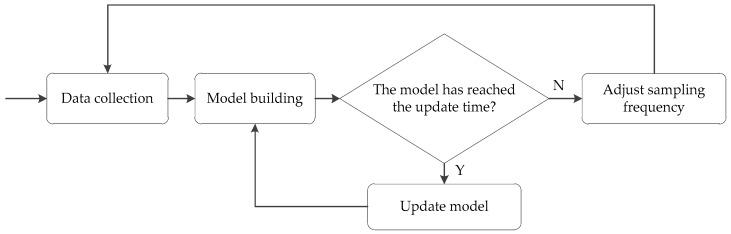
The illustration of the establishment of acquisition process.

According to the time correlation of the measured data collected by the same sensor over a period of time, a linear regression model of the sampling value is constructed to fit the perception data. The method of dynamically updating the model is adopted to make the sampling model meet the requirements of the current adaptive adjustment strategy of the collected data and maintain the timeliness of the model. The adaptive sampling process is mainly divided into three stages, as shown in Figure 3. The first stage collects data based on the initial sampling interval. The second stage trains the model from the latest collected N sampling points, and the third stage is to estimate whether the model has reached the update time. When the model does not reach the update time, the sampling frequency is adjusted according to the model’s estimation results. When the model reaches the update time, it returns to the second stage to rebuild the model.

#### 3.2.2. Fitting of Regression Curve

**Figure 4:**
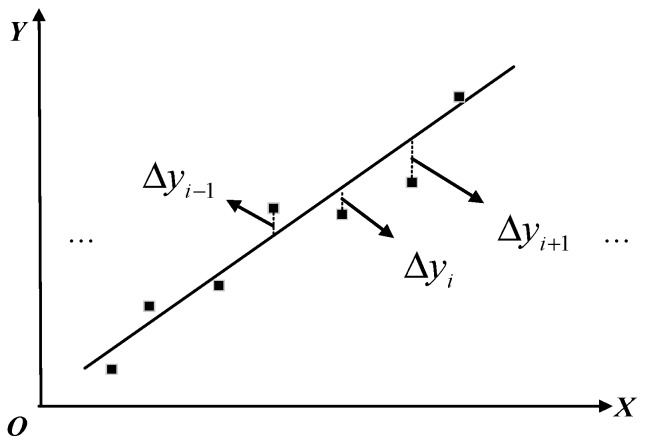
Linear fitting.

Assuming a linear relationship between two physical quantities, the functional form can be written as: y=αx+β, which is called a linear regression equation. The constant α and β are called a linear regression coefficient. However, the actual collected data does not always strictly satisfy the linear characteristics. When the data of each group is substituted y=αx+β, the two sides are not equal. The data points cannot be accurately placed on the straight line corresponding to the formula when drawing, as shown in the Figure 4:

The difference between the actual value and the fitted value of y is defined as the fitting error or residual $\Delta y_{i}$, and the sum of squares of all fitting errors is added to obtain the sum of squares of errors. The best fitting line is also the line that minimizes the error sum of squares. The principle of evaluation extremum value is used to convert the problem of finding the best fit straight line into the smallest square of the error.

Assuming that y=αx+β, if there are *N* actual calibration test points, the residual between the i-th calibration data and the corresponding value on the fitted line is:

(1) $$V_{i}=\Delta y_{i}=y_{i}-(\alpha x_{i}+\beta )$$

The principle of fitting a straight line by least squares is to make $\sum V_{i}^{2}$ the minimum value. The first-order partial derivative of $\sum V_{i}{}^{2}$ to α and β is equal to 0 and finding the expressions of α and β, where y=αx+β is the best fit curve$,\bar{x}=\overset{i-1}{\underset{k=i-N}{\sum }}x_{k},\;\bar{y}=\overset{i-1}{\underset{k=i\;-\;N}{\sum }}y_{k}$

(2) $$\alpha_{1}=\frac{\sum_{k=i-N}^{i-1}\left(x_{k}-\bar{x})(y_{k}-\bar{y}\right)}{\sum_{k=i-N}^{i-1}{\left(x_{k}-\bar{x}\right)}^{2}}$$

(3) $$\beta_{1}=\bar{y}-\alpha_{1}\bar{x}$$

Within a certain time interval, the relationship between the sampling value and the sampling time can be approximately expressed as a linear relationship. By using this relationship, a linear fitting line is established between the sampling value and the sampling time, which is used to calculate and adjust the sampling frequency.

#### 3.2.3. Adaptive Sampling Strategy

This section adjusts the sampling frequency based on the adaptive sampling strategy [27]. Firstly, it calculates the linear median jitter sum based on the straight line fitted by the linear regression.$\;x_{i}$ is used to represent the time of the next sampling point. $x_{i-1}$,$x_{i-2}$,$x_{i-3}$…$x_{i-N}$ represents the sampling time of the first N sampling points. $y_{i-1}$,$y_{i-2}$,$y_{i-3}$…$y_{i-N}$ represents the corresponding sampling value. $y=\alpha_{1}x+\beta_{1}$ represents the straight line fitted by N sampling points. The jitter of the sampling point is represented by the difference from the sampling point to the Y-axis of the median value of the line. The sum of the jitter size of N sampling points relative to the median value of the fitting line is called the linear median jitter sum Ω.

(4) $$\Omega =\sum_{k=i-N}^{i-1}\left|\beta_{1}+\alpha_{1}(\frac{x_{i-N}+x_{i-1}}{2})-y_{k}\right|$$

In the result of linear regression fitting, the value of the linear median jitter sum Ω represents the change trend of the sampling object, which reflects the change speed of the variable of the curve at this point.

**Figure 5:**
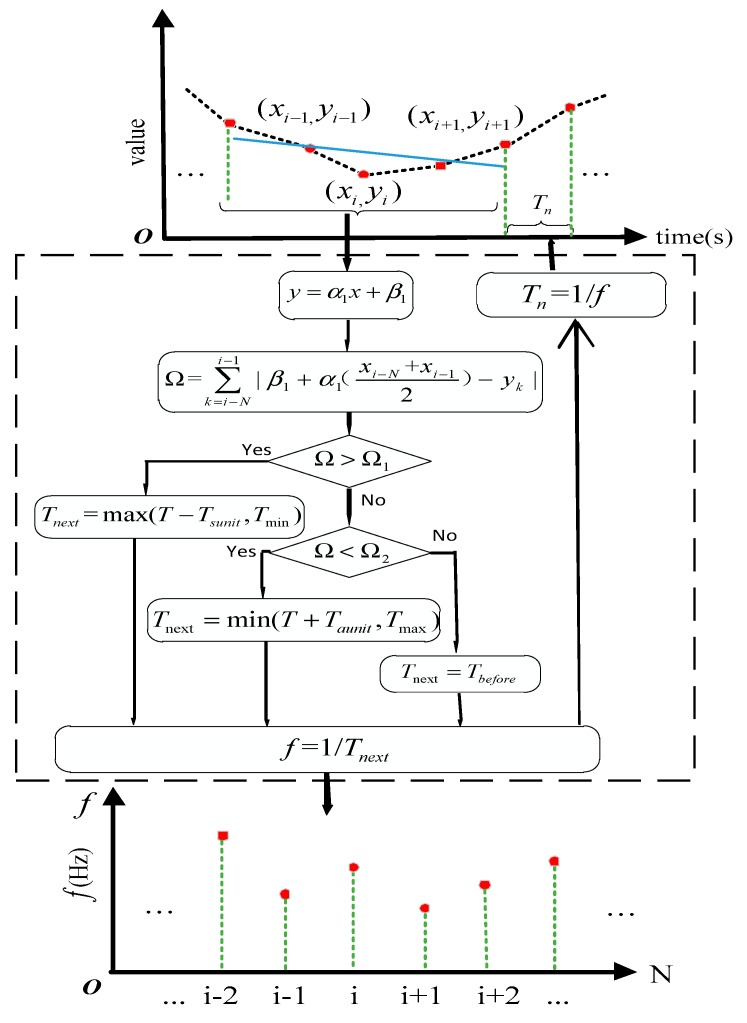
Adaptive sampling strategy.

The adaptive sampling strategy is shown in Figure 5. The sampling frequency is adjusted according to the adaptive sampling strategy. When initialized: $T_{0}=(T_{max}+T_{min})/2$, where $T_{max}$ is the maximum sampling time interval of the sampling object; $T_{min}$ is the minimum sampling time interval of the sampling object; T is the current sampling interval; $T_{next}$ is the next sampling interval; $T_{aunit}$ and $T_{sunit}$ are the unit sampling interval of the increase and decrease to adjust sampling frequency. The $T_{next}$ can be calculated by:

(5) $$T_{next}=\left\{ \begin{array}{cc} \max(T-T_{sunit},T_{\min}) & \Omega >\Omega_{1}\\ \min(T+T_{aunit},T_{\max}) & \Omega <\Omega_{2}\\ T_{\mathrm{before}} & \Omega_{1}\leq \Omega \leq \Omega_{2} \end{array}\right.,$$

The larger Ω is, the greater jitter variation of the sampling object in the sampling process. $\Omega_{1},\Omega_{2}$ represent the threshold of the linear median jitter sum and $\Omega_{2}<\Omega_{1}$. In the formula, $\Omega >\Omega_{1}$ represents the change increase of the sampling object in unit time; $\Omega <\Omega_{2}$ represents the change decrease of the sampling object in unit time; $\Omega_{2}<\Omega <\Omega_{1}$ indicates that the degree of change of the sampling object is not significantly different from the previous time.

It should be noted that the unit sampling time interval of increasing and the unit sampling time interval of decreasing are two independent system parameters, and the values should be different. Generally, in order to improve the accuracy of the sampling fitting curve, the unit sampling time interval of increase $T_{aunit}$ is set to less than the unit sampling time interval of decrease $T_{sunit}$. In this way, it is more cautious to decrease the sampling frequency when the data collection system determines the sampling object changes gently to avoid loss of the information of the sampling object during the sampling process. If the system determines that the sampling object parameters are abrupt, than the sampling frequency will be obviously increase to enhance the corresponding speed of the system for such data changes. By setting the two system parameters to be different, the sensitivity of the adaptive sampling method to the sharp change of the sampling object is improved and the distortion of the sampling object during the sampling process of the edge data acquisition platform is reduced.

## 4. Case Study

**Figure 6:**
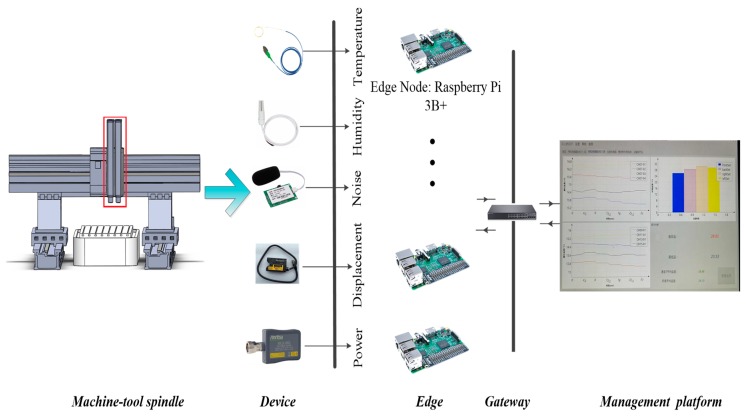
Edge data collection platform.

An edge data collection platform is built in this section, as shown in Figure 6. Our experimental environment located in a machine tool workshop and the research object was the machine-tool spindle. In the experiment, we compared the method proposed in this paper with constant sampling frequency, spectrum-based adaptive method and parameter fluctuations adaptive method in three aspects: Sampling distortion, edge data redundancy and energy consumption. Various sensors were connected by the Raspberry Pi 3B+ [28]. Raspberry Pi 3B+ devices were used as the edge data acquisition node of this experiment. The data-driven adaptive sampling method based on edge computing proposed in this paper was deployed on the Raspberry Pi 3B+. The data collected through Modbus protocol. Modbus [29] was used as the communication protocol between the physical environment and the network environment. The data was stored in InfluxDB [30] database. InfluxDB is a time series database that was deployed as a container [31].

The types and number of sensors used in this experiment are shown in Table 2. Five types of data were collected in the experiment: Temperature, humidity, sound, displacement and power. There were 21 sensor nodes in the experiment. For example, there were 16 temperature nodes in the experiment. Each node was connected to 8 temperature sensors, so there were 128 temperature sensors.

In the experiment, we collected data for eight hours. A total of 3974400 pieces of raw data were collected. The number of data of various types of sensors is shown in Table 3. The raw data includes temperature, humidity, sound, displacement and power which serve as the data source to verify the effectiveness of the data-driven adaptive sampling method.

### 4.1. Improvement of Sampling Distortion

When collecting data in the traditional industrial site, a constant sampling frequency was generally set according to the physical characteristics of the sampling object. However, the equal time interval data collection methods can easily cause the loss of sampling object information: When the sampling object changes rapidly, the sampling information was lost due to the low sampling frequency at the current moment, causing the quality of the original data to be severely reduced.

**Figure 7:**
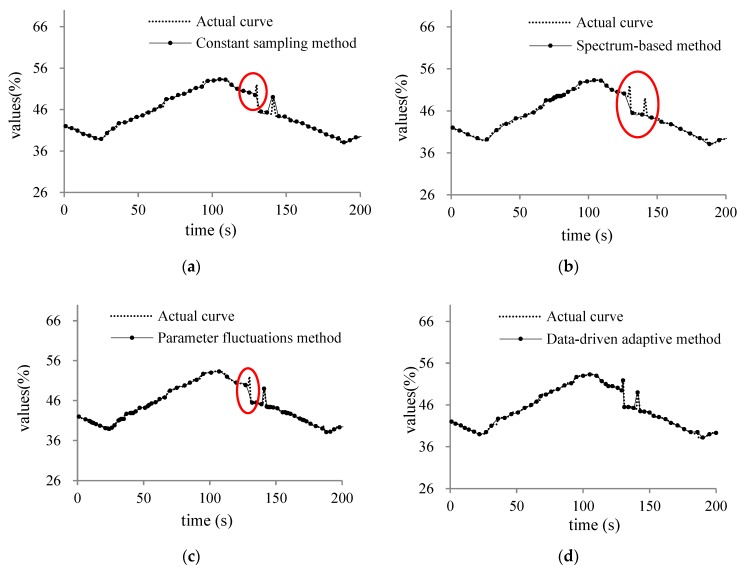
(**a**) constant sampling curve of humidity; (**b**) spectrum-based curve of humidity; (**c**) parameter fluctuations curve of humidity; (**d**) data-driven adaptive curve of humidity.

**Figure 8:**
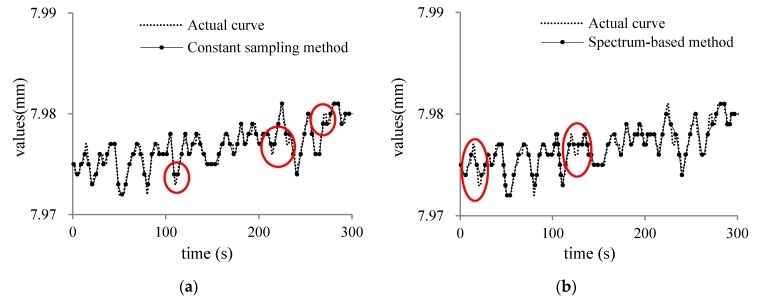

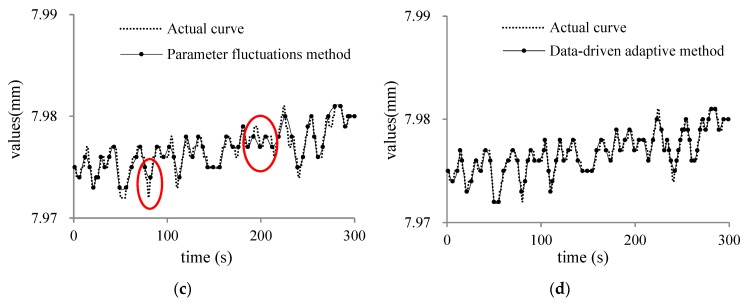
(**a**) constant sampling curve of displacement; (**b**) spectrum-based curve of displacement; (**c**) parameter fluctuations curve of displacement; (**d**) data-driven adaptive curve of displacement.

**Figure 9:**
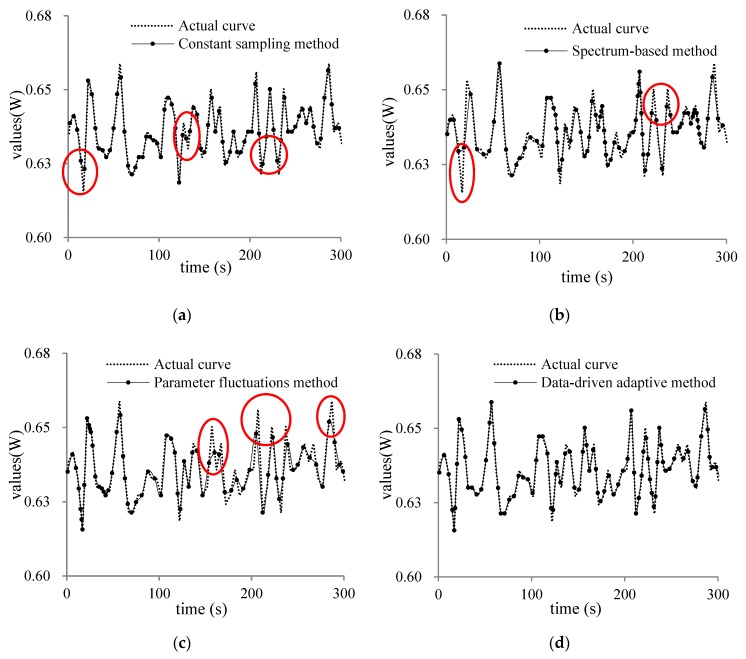
(**a**) constant sampling curve of power; (**b**) spectrum-based curve of power; (**c**) parameter fluctuations curve of power; (**d**) data-driven adaptive curve of power.

In order to prove that the proposed method can effectively improve the sampling distortion, we compared the constant sampling frequency method (T = 4 s), spectrum sensing adaptive method, data fluctuation adaptive method and the data-driven adaptive sampling method proposed in this paper for three types of humidity, displacement and power sensor. The three sampling objects results are shown as Figure 7, Figure 8 and Figure 9.

In Figure 7, Figure 8 and Figure 9a is the traditional sampling curve with constant sampling frequency (T = 4 s); (b) is the adaptive sampling curve with spectrum-based method; (c) is the adaptive sampling curve with parameter fluctuations adaptive method; (d) is the sampling curve using the adaptive sampling method proposed in this paper. The location of the loss of sampling information caused by the traditional constant sampling frequency is shown with red circle in the figure. According to the sampling curve, we can find that the sampling curves obtained by the adaptive sampling method proposed in this paper can better restore the actual change curve of the sampling object and the sampling curve is closer to the actual change of the sampling object. The change trend of the sampling object at the sensitive moment can be observed more clearly by adjusting the sampling frequency adaptively at the edge node.

### 4.2. The Edge Data Redundancy

**Figure 10:**
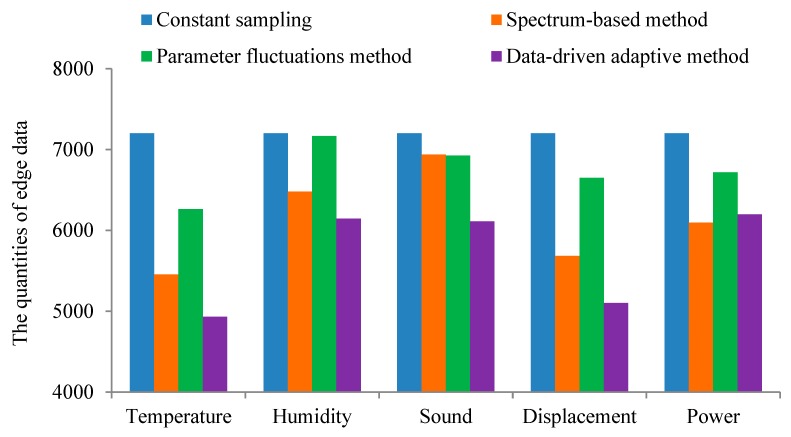
The quantities of edge data.

At present, the traditional data acquisition system mostly adopts the data acquisition scheme of equal interval, while some acquisition systems with high processing efficiency hope to reduce the storage space of data. The use of the traditional equal-interval data sampling method tends to lead to an extreme case: When the information of the sampling object changes slowly, a large amount of redundant data was collected due to the small data acquisition interval which will take up a large amount of storage space and seriously degrade the quality of original data. In order to prove the proposed method can effectively improve the edge data redundancy, we used the constant sampling frequency method (T = 4 s), spectrum-based adaptive method, parameter fluctuations adaptive method and the data-driven adaptive sampling method proposed in this paper for five types of sensors: Temperature, humidity, sound, displacement and power. We observed the amount of edge data collected of four sampling methods. The edge data volume of temperature, humidity, sound, displacement and power data under different sampling strategies are shown in Figure 10.

It can be seen from Figure 10 that all kinds of adaptive sampling methods can reduce the degree of edge data redundancy compared with traditional constant sampling frequency methods. The method proposed in this paper is more effective than other adaptive sampling methods in edge data redundancy. Compared with constant sampling frequency, the quantities of edge data for temperature, humidity, sound, displacement and power data collection decreased by 31.5%, 14.67%, 15.11%, 29.14% and 13.92%, respectively.

Since the data of the temperature sensor is the main data source in this experiment, we analyzed the data of the 16 temperature collection nodes in detail. In order to prove the universal applicability of our method, we divided the eight sensors of each temperature sensor node into four groups. We used the constant sampling frequency method (T = 4 s), spectrum-based adaptive method, parameter fluctuations adaptive method and the data-driven adaptive sampling method on four groups of temperature sensors to collect data. Eight hours of collected data were recorded. The data volume of four different sampling methods in each temperature collection node and the reduction in data compared to the constant sampling frequency are shown in Table 4:

It can be found from Table 4 that the adaptive sampling method proposed in this paper can reduce the temperature data by at least 18.97% and at most by 36.05%. The data-driven adaptive sampling method based on edge computing is more effective than other adaptive methods. Compared with the traditional constant sampling frequency method, the data-driven adaptive sampling method proposed in this paper can reduce the temperature data redundancy by an average of 31.45%.

### 4.3. The Energy Consumption

In the monitoring of industrial environment, the energy consumption caused by data collection should also be taken seriously. The energy consumption of unnecessary data collection does not bring any economic benefits. In industrial field data collection, due to some special circumstances, some battery-powered sensing devices will be used. For such battery-powered sensors, energy consumption from data collection is the main way to affect service time. Adaptive sampling can reduce unnecessary data collection and unnecessary energy consumption which extends the service time of battery-powered sensing devices. In order to roughly calculate the energy consumption of the sensor, we unified the power supply voltage of all sensors to 5 V, the corresponding time is 1/e (e = 2.718281828…), the power consumption of the measurement is 1.5 mA and the power consumption of the dormant is 10 uA according to the relevant information. In the experiment, the power consumption of the dormant has little effect on the results of this experiment, so the energy consumption caused by the sensor dormancy is ignored. The energy consumption of the data collected by the sensor once can be roughly estimated to be:

(6) $$5\times \frac{1.5}{1000}\times \frac{1}{e}=0.00276=2.76\times 10^{-3}J$$

**Figure 11:**
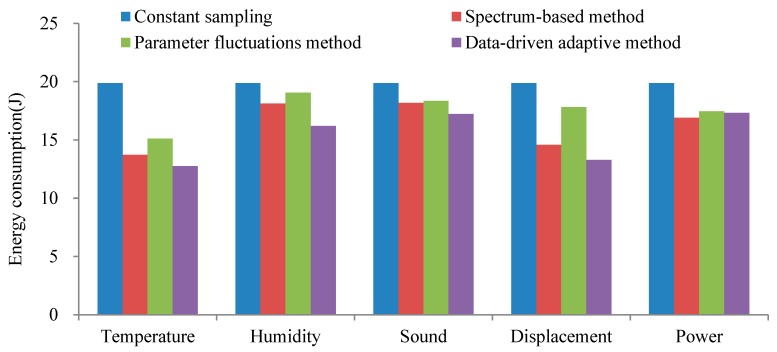
The energy consumption.

We respectively used the constant sampling frequency method (T = 4 s), spectrum-based adaptive method, parameter fluctuations adaptive method and the data-driven adaptive sampling method proposed in this paper on five types of sensors. We compared and observed the energy consumption caused by the four sampling strategies applied to the sensors for 8 h. The energy consumption of data collection for temperature, humidity, sound, displacement and power under different sampling strategies as presented in Figure 11:

It can be seen from Figure 11 that the data collection energy consumption by the constant sampling frequency method is relatively larger. Although the spectrum-based adaptive sampling method and the parameter fluctuations adaptive sampling method can reduce energy consumption, the method proposed in this paper is the best in terms of energy consumption. By deploying a data-driven adaptive sampling method at the edge, the data collection energy consumption of different sampling objects can be reduced. Compared with the traditional constant sampling frequency method, the energy consumption for temperature, humidity, sound, displacement and power sensors decreased by 35.83%, 18.47%, 13.33%, 33.14% and 12.86%, respectively.

## 5. Conclusions

For the problem of sampling distortion, edge data redundancy and energy consumption caused by constant sampling frequency of edge devices under the IIoT, a data-driven adaptive sampling method based on edge computing is proposed in this paper. The linear median jitter sum and adaptive sampling strategy are used to adjust the sampling frequency. The constant sampling frequency method (T = 4 s), spectrum-based adaptive method, parameter fluctuations adaptive method and the data-driven adaptive sampling method proposed in this paper were compared and observed, respectively, in terms of sampling distortion, edge data redundancy and energy consumption. Compared with constant sampling, the results show the proposed method can reduce the edge data redundancy by more than 13.92% and the energy consumption by more than 12.86%. The proposed method is more effective than other adaptive sampling methods. By applying the method proposed in this paper to the sensors (temperature, humidity, noise, displacement and power sensors), the sampling distortion, edge data redundancy and energy consumption can be effectively reduced.

Possible future work will consider an adaptive sampling method for high-speed and real-time data acquisition. Due to the resource limitation of edge nodes, the flexibility of adaptive sampling method can also be improved according to the real-time calculation of resource availability of edge nodes to maximize the resource utilization of edge nodes.

## Figures and Tables

**Table 1:** Comparison of methods.

	Spectrum-Based	Parameter Fluctuations	Data-Driven
Accuracy	**√**		**√**
Rapid response		**√**	**√**
Feasibility of edge device		**√**	**√**

**Table 2:** Types and number of sensors.

	Number of Nodes	Number of Sensors in a Single Node	Total
Temperature sensor	16	8	128
Humidity sensor	2	1	2
Sound sensor	1	2	2
Displacement sensor	1	3	3
Power sensor	1	3	3

**Table 3:** Number of sensor data.

	Temperature	Humidity	Sound	Displacement	Power
Number	3686400	57600	57600	86400	86400

**Table 4:** Comparison of data collected by various methods.

**Node**	**1**	**2**	**3**	**4**	**5**	**6**	**7**	**8**
Constant sampling	14400	14400	14400	14400	14400	14400	14400	14400
Spectrum-based	9984	9874	10122	9857	10063	10008	9992	10206
Parameter fluctuations	11809	11058	10868	11399	12276	11752	11625	11314
Data-driven	9648	9254	9462	9545	10636	10502	9292	9440
Decrease	33.00%	35.74%	34.29%	33.72%	26.14%	27.07%	35.47%	34.44%
**Node**	**9**	**10**	**11**	**12**	**13**	**14**	**15**	**16**
Constant sampling	14400	14400	14400	14400	14400	14400	14400	14400
Spectrum-based	11086	11321	11632	10012	9971	10282	9521	9597
Parameter fluctuations	12541	12360	12888	12932	11187	12406	12254	12030
Data-driven	11089	10049	11669	9921	9310	9697	9215	9209
Decrease	22.99%	30.22%	18.97%	31.10%	35.35%	32.66%	36.01%	36.05%
